# Sepsis following acute pyelonephritis caused by *Trueperella bernardiae*: a case report and literature review

**DOI:** 10.1186/s12879-023-08080-8

**Published:** 2023-02-24

**Authors:** Yuki Matsuhisa, Tsuneaki Kenzaka, Shinichiro Kobayashi, Jun Taguchi, Hideo Hirose, Tadao Gotoh

**Affiliations:** 1 Department of General Medicine, Center for Community Medicine in North-Western Gifu Prefecture National Health Insurance Shirotori Hospital, 1205-1, Tamezani, Shirotori-Cho, Gujo, Gifu 501-5122 Japan; 2 Department of Pediatrics, Center for Community Medicine in North-Western Gifu Prefecture National Health Insurance Shirotori Hospital, 1205-1, Tamezani, Shirotori-Cho, Gujo, Gifu 501-5122 Japan; 3 Department of Internal Medicine, Hyogo Prefectural Tamba Medical Center, 2002-7 Iso, Hikami-Cho, Tamba, Hyogo 669-3495 Japan; 4 Division of Community Medicine and Career Development, Kobe University Graduate School of Medicine, 2-1-5 Arata-Cho, Hyogo-Ku, Kobe, Hyogo 652-0032 Japan; 5 Department of Infectious Disease, Gifu Prefectural General Medical Center, 4-6-1, Noisshiki, Gifu-shi, Gifu 500-8717 Japan

**Keywords:** *Trueperella bernardiae*, Sepsis, Acute pyelonephritis, Bedridden, Diaper using

## Abstract

**Background:**

*Trueperella bernardiae* is a coryneform, gram-positive bacterium that is a commensal of the skin and upper respiratory tract. It is treated as a contaminant and rarely causes infections. Blood, urine, and abscesses have been previously reported as the most common sites of infection. Infections caused by *T. bernardiae* are rarely reported in bedridden very old patients with reduced activities of daily living (ADL). In this report, we describe a case of sepsis due to acute pyelonephritis caused by *T. bernardiae* in a very old patient with impaired ADL.

**Case presentation:**

A 94-year-old woman had a home visit from her local physician. She was bedridden and used diapers. On the day of admission, she presented with fever and dyspnea and was admitted with a diagnosis of sepsis associated with acute pyelonephritis. *T. bernardiae* was detected in blood and urine cultures; furthermore, multiple bacteria were detected in a urine culture. She was treated with ampicillin/sulbactam 3 g every 12 h on the day of admission. The fever was controlled, and inhaled oxygen 1 L/min via a nasal cannula was administered for dyspnea until hospitalization day 2. On hospitalization day 2, her fever resolved to 36 °C. Antimicrobials were de-escalated and changed to cephazolin and then to cephalexin on hospitalization days 9 and 16, respectively, and were continued until day 22. On hospitalization day 28, the urinary tract infection flared up; however, her fever resolved by hospitalization day 38 after the re-administration of antimicrobial agents. She was discharged on hospitalization day 60.

**Conclusions:**

We encountered a rare case of sepsis following acute pyelonephritis caused by *T. bernardiae* infection. When bedridden, diaper-using, very old patients present with urinary tract infections caused by multiple bacteria, the presence of rare opportunistic organisms, such as *T. bernardiae*, should be considered.

## Background

*Trueperella bernardiae* is a coryneform, gram-positive coccobacillus [1, 2]. Until 1995, *T. bernardiae*, which is characterized by negative catalase production, belonged to Centers for Disease Control and Prevention coryneform group 2 [1]. It was assigned to the genus *Actinomyces* in 1995 [2], *Arcanobacterium* in 1997 [3], and transferred to the genus *Trueperella* in 2011 [4]. *T. bernardiae* is a commensal bacterium found on the skin and in the upper respiratory tract. It is usually treated as a contaminant and rarely as an infectious agent [1]. Although reports on *T. bernardiae* infection have been increasing [5–7], there have been few reports in bedridden old patients with impaired activities of daily living (ADL) [7, 8]. In this report, we describe a case of sepsis due to acute pyelonephritis caused by *T. bernardiae* in a very old patient with impaired ADL.

## Case presentation

A 94-year-old woman was receiving home visits and treatment for comorbidities including hypertension, chronic heart failure, dementia, chronic kidney disease, osteoporosis, and constipation. She sustained a left femoral transverse fracture 4 years and 2 months before her presentation and has been bedridden since then. She was attended to at home by a house call physician because of dyspnea on the day of admission and was then rushed to our hospital because of fever (40 °C) with chills. According to her medical history, she had suffered a spinal compression fracture at the age of 84 years and a left femoral transverse fracture at the age of 90 years, for which she underwent artificial head replacement surgery. She was bedridden and used diapers for excretion.

The vital signs at the time of our visit were as follows: level of consciousness, Glasgow Coma Scale (GCS) score, 11 points (E3V2M6); body temperature, 40 °C; blood pressure, 97/56 mmHg; heart rate, 110 beats/min; respiratory rate, 30 breaths/min; and SpO_2_, 96% (O_2_ mask 6 L/min). Pupils were of the same size, normal contralateral light reflex was noted, and there was no pharyngeal redness; no tonsillar redness, swelling, or moss white color. The heart and respiratory sounds were normal. There was no skin rash or abnormal findings, such as tenderness, heat, or swelling of the joints. She tended to be somnolent and had no clear costovertebral angle tenderness.

**Fig. 1:**
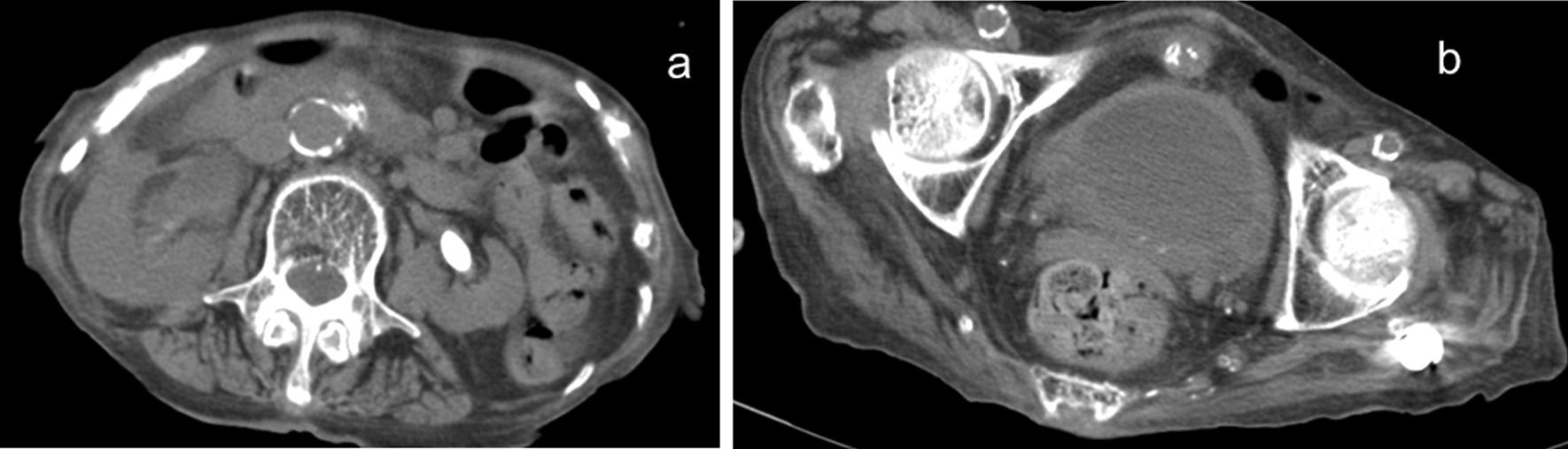
Abdominal computed tomography at admission. There are stones in both kidneys, right ureteral dilation (**a**), and a thickened bladder wall (**b**)

Laboratory data at the time of her visit are presented in Table 1. The white blood cell (WBC) count was 17,400/μL and the C-reactive protein (CRP) level was 12.31 mg/dL. Urinalysis showed a leukocyte count of > 100/high power field, gram-positive cocci (3+), gram-positive rods (1+), and gram-negative rods (3+) on urine Gram staining. The Quick Sequential Organ Failure Assessment (qSOFA) score was 3 points [9, 10] and the SOFA score was 3 points [10–12]. Abdominal computed tomography showed stones in both kidneys, right ureteral dilation, and a thickened bladder wall (Fig. 1).

**Table 1:** Patient’s laboratory data on admission

Parameter	Recorded value	Standard value
White blood cell count	11,260/µL	4500–7500/µL
Neutrophils	90.7%	42–74%
Lymphocytes	5.3%	18–50%
Monocytes	4.0%	1–10%
Hemoglobin	9.6 g/dL	11.3–15.2 g/dL
Platelet count	36.7 × 104/µL	15.0–35.0 × 104/µL
C-reactive protein level	12.31 mg/dL	0.00–0.30 mg/dL
Blood urea nitrogen level	41.3 mg/dL	8.0–20.0 mg/dL
Creatinine level	1.85 mg/dL	0.40–0.80 mg/dL
Total bilirubin	0.5 mg/dL	0.2–1.2 mg/dL
PaO_2_	221 mmHg	80–100 mmHg
FiO_2_	0.5	0.21
PaO_2_/FiO_2_	442	> 500

She was diagnosed as having sepsis associated with acute pyelonephritis. Gram-positive cocci were detected in the urine, and penicillin antimicrobials were selected to cover enterococci. Intravenous ampicillin/sulbactam administration of 3 g every 12 h was initiated on the day of admission. The fever was controlled, and inhaled oxygen 1 L/min using a nasal cannula was administered for dyspnea until hospitalization day 2. The temperature temporarily dropped to the 37 °C range but rose again to 40 °C, followed by remittent fever. The temperature resolved to 36 °C on hospitalization day 2. The level of consciousness also improved to a GCS score of 15 points (E4V5M6). The number of viable bacteria in a urine culture was 10^6^ CFU/mL, and *Pseudomonas *sp*.* (3+), *Streptococcus *sp. (3+) and *Corynebacterium *sp. (1+) were detected. Since ampicillin/sulbactam was effective, *Pseudomonas *sp. was considered not to be the initiating bacterium. On hospitalization day 9, based on the antimicrobial susceptibility of other bacteria, the patient was started receiving cefazolin 1 g intravenous infusion every 12 h. On hospitalization day 10, gram-positive coccobacilli, identified as *Arcanobacterium* sp, were detected in two sets of blood cultures. The samples were analyzed using a Matrix Assisted Laser Desorption/Ionization Time of Flight Mass Spectrometer (MALDI-TOF MS) and were identified as *T. bernardiae* (identification score > 2.0). Similarly, *T. bernardiae* was detected in a urine culture by MALDI-TOS MS analysis. 16SrRNA gene sequencing was not performed.

As no *T. bernardiae* breakpoints are specified by the Clinical and Laboratory Standards Institute (CLSI) or The European Committee on Antimicrobial Susceptibility Testing (EUCAST), susceptibility testing was performed based on the CLSI criteria for *Corynebacterium *sp., and the susceptibility was determined based on the size of the inhibition circle [13]. Susceptible and resistant bacteria were determined based on whether the inhibition circle was > 30 mm using the disk method. Antimicrobial agents were also selected based on this method. The results are presented in Table 2. The bacteria were susceptible to penicillin G, amoxicillin, piperacillin, cefazolin, cefotiam, ceftriaxone, cefozopran, meropenem, imipenem/cilastatin, amoxicillin/clavulanic acid, piperacillin/tazobactam, amikacin, erythromycin, clarithromycin, clindamycin, minomycin, fosfomycin, and vancomycin, but were resistant to levofloxacin and sulfamethoxazole/trimethoprim. Bacteria from urine and blood cultures were similarly susceptible. *T. bernardiae* was susceptible to cephazolin and, therefore, the antimicrobial therapy was continued.

**Table 2:** Results of drug sensitivity test for *Trueperella bernardiae* cultured

Disk method			
Antibiotic	Sensitivity	Antibiotic	Sensitivity
Penicillin G	S	Piperacillin/tazobactam	S
Amoxicillin	S	Amikacin	S
Piperacillin	S	Erythromycin	S
Cefazolin	S	Clarithromycin	S
Cefotiam	S	Clindamycin	S
Ceftriaxone	S	Minomycin	S
Cefozopran	S	Fosfomycin	S
Meropenem	S	Vancomycin	S
Imipenem/cilastatin	S	Levofloxacin	R
Amoxicillin/clavulanic acid	S	Sulfamethoxazole/trimethoprim	R

The “S” and “R” represent “susceptible” and “resistant,” respectively

**Fig. 2:**
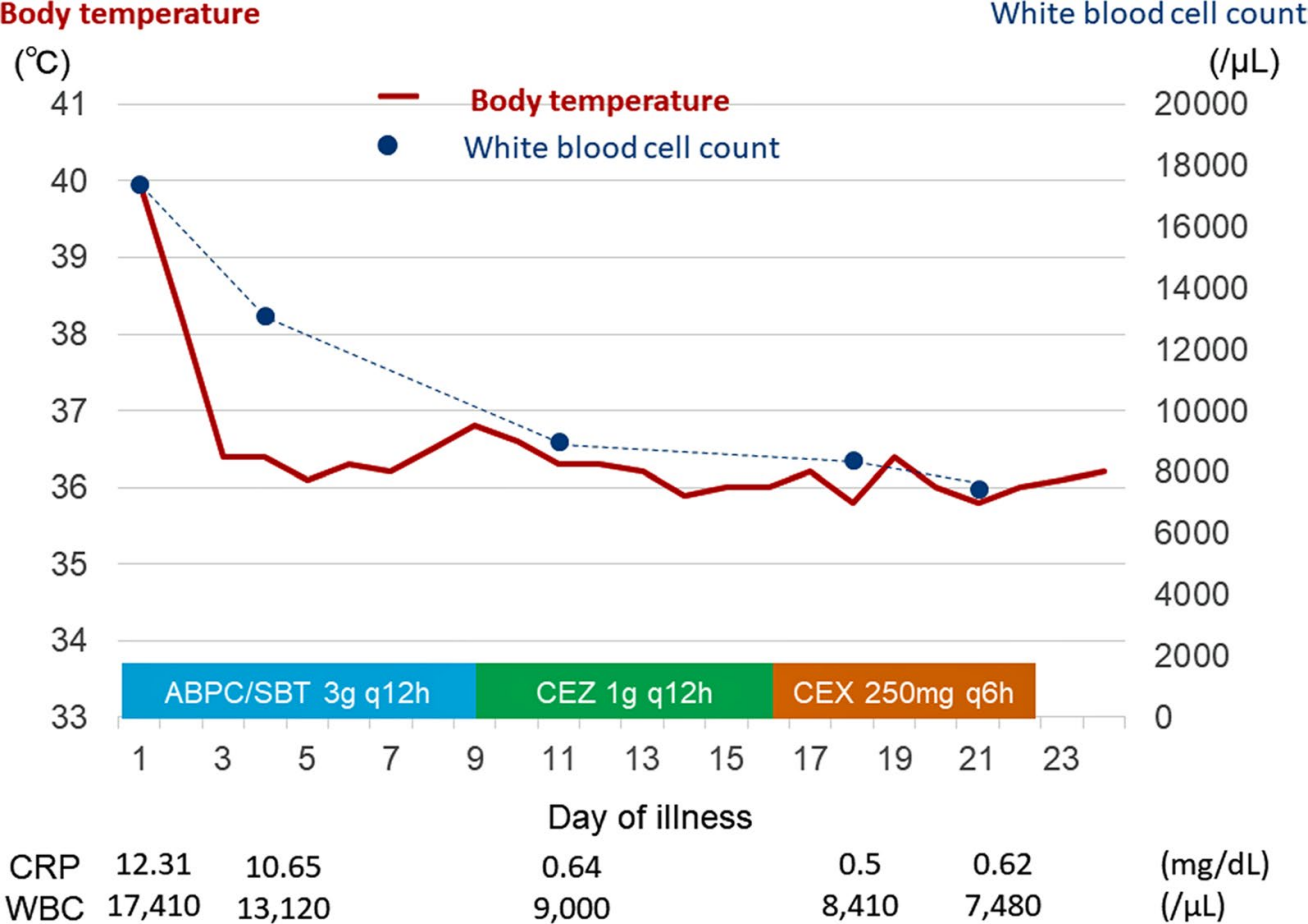
Clinical course after treatment initiation. ABPC/SBT: ampicillin/sulbactam; CEZ: cefazolin; CEX: cephalexin

Two sets of blood samples cultured on hospitalization day 11 were negative. Since oral intake was improved, she was switched to oral cephalexin 1 g/day on hospitalization day 16. On hospitalization day 22, antimicrobial therapy was terminated (Fig. 2).

Thereafter, rehabilitation is provided for declining ADLs and she was discharged on hospitalization day 60.

## Discussion

We encountered a case of sepsis following acute pyelonephritis caused by *T. bernardiae*, a commensal bacterium found on the skin and in the upper respiratory tract. Although *T. bernardiae* is a rare cause of infection, it was detected in two sets of blood cultures and in a urine culture in the present case. Moreover, it was observed to be the causative agent of the infection, which was considered to have occurred because the patient was very old, bedridden with reduced ADL, and used diapers for defecation.

In this case, multiple bacteria were initially detected in a urine culture. As ampicillin/sulbactam was effective, *Pseudomonas *sp. was not considered for treatment. Other detected bacteria were sensitive to ampicillin/sulbactam and cefazolin. Finally, only *T. bernardiae* was detected in blood cultures and was determined to be the initiating bacterium for sepsis. It seems possible that the urinary tract was also infected with several other bacteria.

In general, we do not routinely keep images in our daily clinical practice. Unfortunately, the Gram staining and culture results in this case were not recorded because there is no laboratory in the hospital to obtain the information, and the results were outsourced to another laboratory outside of the hospital.

Regarding the antimicrobial susceptibility of *T. bernardiae*, resistance to penicillins [14, 15], aminoglycosides [14], macrolides [16–19], quinolones [19], sulfamethoxazole/trimethoprim [14, 18], and fosfomycin [8] have been reported. In this case, the patient was non-responsive to quinolones and sulfamethoxazole/trimethoprim. No cephalosporin-resistant bacteria have been reported.

As there are no reports of *T. bernardiae* resistance to β-lactamase-containing penicillin, carbapenems, and cephems (although resistance to penicillin has been reported), we suggest that before the results of susceptibility testing are known, antimicrobial therapy should include β-lactamase-containing penicillin, if the patient is in good general condition, and carbapenems, if the patient is in poor general condition.

As reports of infections caused by *T. bernardiae* are rare, a comprehensive literature review was conducted. Thirty articles were searched using the keywords "*Trueperella bernardiae*," "*Arcanobacterium bernardiae*," or "*Actinomyces bernardiae*" on PubMed database. The search identified 19 case reports (including 27 cases) since 1996 (Table 3). In addition, five case reports (including five cases) related to these articles were not archived in PubMed [5–8, 14–33]. (Last PubMed search: November 28, 2022).

**Table 3:** Clinical features of cases of *Trueperella bernardiae* infection in the literature

Case	Study	Reference number	Age (years)/sex	Localization of infection	Clinical manifestation	Clinical setting	Other co-isolated organisms	Treatment (period)	Immunocompromised status	Outcome
1	Ieven et al., 1996, Belgium	[20]	69/M	Urinary tract	Fever	Neurogenic bladder dysfunction	NA	Ciprofloxacin (4 days), amoxicillin/clavulanic acid (13 days)	NA	Cure
2	Lepargner et al., 1998, France	[21]	75/M	Urinary tract	Right back pain, fever	After treatment for urothelial carcinoma without ganglionic metastases	NA	Netilmicin + cefixime (NA), amoxicillin (NA)	NA	Cure
3	Adderson, 1998, USA	[22]	19/F	Hip effusion	Right hip and knee pain, fever, deteriorating renal function	4-year use of corticosteroids and cyclophosphamide for lupus nephritis	None	Piperacillin/tazobactam + gentamicin (2 days), vancomycin + cefotaxime (2 days), clindamycin (6 weeks)	Corticosteroids and cyclophosphamide use	Cure
4	Bemer et al., 2009, France	[23]	63/M	Knee joint wound	Wound drainage	Tuberculous arthritis of the knee, 6-year-old	*Staphylococcus aureus*	Clindamycin + fusidic acid (3 weeks)	NA	Cure
5	Loiez et al., 2009, France	[14]	78/M	Hip prosthesis	Hematoma, pain	Left total hip prosthesis 27 years ago	NA	Linezolid + cefotaxime (NA), rifampicin + olfloxiacin (12 weeks)	NA	Cure
6	Clarke et al., 2010, USA	[24]	62/F	Abdominal wall skin abscess	Left lower abdominal pain, redness of the skin, fever, mass on the skin	Diabetes mellitus type 2, obesity, sleep apnea	*Morganella morganii*	Vancomycin + aztreonam (NA), Piperacillin/tazobactam (NA)	Diabetes mellitus, HbA1c: NA	Cure
7	Sirijatuphat et al., 2010, Thailand	[25]	60/M	Kidney abscess, Perinephric abscess	Fever, dysuria, loin pain, weight loss	Diabetes mellitus and renal stones	NA	Ceftazidime (NA), ceftriaxone + clindamycin (NA), clindamicyn (3 months)	NA	Cure
8	Weitzel et al., 2011, Chile	[26]	72/F	Sacral ulcer, Blood	Fever, chills, anorexia, prostration	Alzheimer disease, chronic pressure ulcers 6 months earlier	Methicillin-resistant* Staphylococcus aureus* *Citrobacter freundii*	Ceftriaxone + metronidazole (6 days), amoxicillin/clavulanic acid (6 weeks)	NA	Cure
9	Otto et al., 2013, France	[8]	78/F	Sacral and leg ulcer, blood	Fever, leg and sacral ulcer	Bedridden, obesity, chronic leg ulcer infection	*Bacteroides fragilis, Enterococcus avium*	Ceftriaxone + cyprofloxacine + metrlnidazole (NA), amoxicillin/clavulanic acid (10 days)	Obesity	Cure
10	Partha et al., 2015, the UK	[27]	68/F	Brain abscess	Confusion, vomiting, slurry speech, incontinence	Diabetes insipidus, rheumatoid arthritis, chronic suppurative otitis media	*Peptoniphilus harei*	Gentamicin + teicoplanin + metronidazole + ceftriaxone + acyclovir (NA), amoxicillin + metronidazole + ceftazidime, ceftriaxione (12 days)	Diabetes mellitus HbA1c: NA, Rheumatoid arthritis	Cure
11	Schneider et al., 2015, Denmark	[28]	45/M	Skin ulcer, blood	Foot ulcers, fever	Diabetic foot gangrene	*Staphylococcus simulans, Peptostreptococcus*	Piperacillin/tazobactam + ciprofloxacin + gentamicin (16 days), amoxicillin (14 days)	Diabetes mellitus HbA1c: NA	Cure
12	Rattes et al., 2016, Brazil	[29]	24/F	Wound	Fever, pain, purulent periumbilical secretion	Periumbilical cellulitis after laparoscopic cholecystectomy	NA	Piperacillin/tazobactam + vancomycin (6 days), amoxicillin/clavulanic acid (7 days)	NA	Cure
13	Gilarranz et al., 2016, Spain	[30]	69/F	Knee prosthesis	Left leg pain	Knee hematoma in a patient who underwent total knee replacement	NA	Ciprofloxacin (2 weeks)	NA	Cure
14	VanGorder et al., 2016, USA	[31]	77/F	Skin abscess	Indurated lesion, pain, drainage	NA	NA	Trimethoprim/sulfamethoxazole (NA)	NA	Cure
15	Kawahara et al., 2017, Japan	[16]	91/F	Urinary tract, blood	Fever, desaturation, shock vital	Parkinson disease, renal stone, hydronephrosis	NA	Meropenem (10 days)	NA	Cure
16	Cobo et al., 2017, Spain	[19]	69/F	Blood	Fever, pain, wound drainage	Laparotomy due to pericolostomy eventration	NA	Metronidazole + ciprofloxacin (7 days), amoxicillin/clavulanic acid (7 days)	NA	Cure
17		[19]	70/F	Purulent exudate	Fever, pain, infection signs in a granuloma	Ulcerated inguinal granuloma in patient with metastatic ovarian cancer	*Escherichia coli*	Metronidazole (7 days), amoxicillin/clavulanic acid (7 days)	Metastatic cancer, chemotherapy	Cure
18	Gowe et al., 2018, USA	[32]	57/M	Olecranon	Fever, pain and swelling in right elbow	Olecranon bursitis in patient after olecranon bursectomy and tenotomy	NA	Ceftriaxone + vancomycin (3 days), doxycycline (14 days)	NA	Cure
19	Lawrence et al., 2018, UK	[17]	56/M	Bone	NA	Diabetic foot infection	NA	NA	Diabetes mellitus HbA1c: NA	NA
20		[17]	42/F	Urine	NA	Ileal conduit	*Enterococcus faecalis*	NA	NA	NA
21		[17]	32/F	Breast abscess	NA	Breast abscess	None	NA	NA	NA
22		[17]	42/M	Bone	NA	Diabetic foot infection	*Enterobacter cloacae, Citrobacter koseri, Corynebacterium *sp*.*	NA	Diabetes mellitus HbA1c: NA	NA
23		[17]	43/M	Blood	NA	Septic thrombophlebitis in injection drug user	*Fusobacterium goindiaformans, Actinomyces funkei*	NA	Injection drug user	NA
24		[17]	50/F	Blood	NA	Metastatic cervical cancer	*Escherichia coli*	NA	Metastatic cancer	NA
25		[17]	32/M	Blood	NA	Still disease	*Fusobacterium goindiaformans*	NA	Still disease	NA
26		[17]	45/M	Blood, vein, left leg abscess	Swellings in left leg and groin, fever, and cough with pleuritic chest pain	Septic thrombophlebitis in injection drug user	*Peptoniphilus harei*	Cefuroxime + metronidazole (2 weeks), amoxicillin/clavulanic acid (4 weeks)	Injection drug user	Cure
27	Calatrava et al., 2019, Spain	[18]	39/F	Breast abscess	Pain and local swelling in right breast	Unremarkable	*Actinotignum sanguinis*	Cloxaxillin (7 days), amoxicillin/clavulanic acid (10 days)	NA	Cure
28	Pan et al., 2019, USA	[33]	5/M	Brain abscess	Left-sided otalgia, lethargy, emesis, and decreased oral intake, fever, seizure	Acute-on-chronic suppurative left otitis media after bilateral percutaneous tympanostomy tube placement	*Actinomyces europaeus, Corynebacterium amycolatum, Corynebacterium aurimucosum*	Piperacillin/tazobactam + ceftriaxone (one dose), vancomycin + cefepime + metronidazole (6 days), Meropenem (7 days), vancomycin + cefepime + metronidazole (6 weeks), amoxicillin (6 months)	NA	Cure
29	Roh et al., 2019, Korea	[15]	83/F	Blood	Fever, hypotension	Diabetes mellitus and cerebrovascular disease	*Staphyrococcus aureus*	Meropenem (2 days), teicoplanin (11 days)	Diabetes mellitus HbA1c: NA	Cure
30	Tang et al., 2021, USA	[5]	71/M	Wound	Bulge on right hip where there is a surgical scar	Hip arthroplasty with hematoma on surgical scar	NA	Doxycycline (7 days), ceftriaxone (6 weeks),	NA	Cure
31	Casale et al., 2022, Italy	[6]	78/F	Blood	Fever, abdominopelvic pain	Total vulvectomy due to a keratinizing squamous infiltrating carcinoma	*Bacteroides fragilis, Enterococcus avium*	Piperacillin/tazobactam + metronidazole (NA), clindamycin + metronidazole (14 days)	Cancer, chemotherapy	Cure
32	Seki et al., 2022, Japan	[7]	80/F	Urinary tract, Blood	Fever, desaturation, disturbed level of consciousness	Post stroke, dementia	*Proteus mirabilis, *Methicillin-resistant* Staphylococcus aureus*	Piperacillin/tazobactam (14 days)	NA	Cure
33	This present case		94/F	Urinary tract, blood	Fever	Bedridden, used diapers	*Pseudomonas *sp*.* *Streptococcus* sp*.* *Corynebacterium *sp*.*	Ampicillin/sulbactam (8 days), Cefazolin (7 days), cefalexin (7 days), cefalexin (3 days), cefazolin (5 days), ampicillin/sulbactam (7 days), amoxicillin/clavulanic acid (18 days)	None HbA1c(NGSP): 5.2%	Cure

NA: not applicable

The median age of the 32 patients (14 men and 18 women) was 62 years (range, 5–91 years), with no obvious age or sex disparity. *T. bernardiae* was often detected in the presence of other organisms, and the prognosis was good in many cases. Among the reported cases, nine, six, four, four, and two cases were of British, American, French, Spanish, and Japanese patients, respectively, while there was one case of Belgian, Chilean, Brazilian, Korean, Italian, Thai, and Danish patient each. The underlying diseases varied from diabetes mellitus to post-arthroplasty but were most frequently identified in patients with an immunocompromised status. The sites of infection included the urinary tract, blood, brain abscess, and breast abscess in five, 12, two, and two cases, respectively, and in other sites (including the skin, surgical sites, and joints). In the present case, the organism was detected in two sets of blood and urine cultures, suggesting that sepsis might have occurred, following the acute pyelonephritis caused by *T. bernardiae.*

The long-term decline in physical activity due to aging, loss of strength and motivation, and various diseases of the musculoskeletal and cardiovascular systems may lead to disuse syndrome [34, 35]. As disuse syndrome progresses, the patient develops secondary disabilities, such as muscle weakness, joint contractures, circulatory disorders, and psychiatric symptoms, such as depression and dementia. As a result, they become susceptible to infection. In addition, diaper users have a high incidence of chronic cystitis [36, 37]. In the present case, the patient was bedridden, very old, and her ADL had decreased, so she could have been easily infected. Since *T. bernardiae* is a commensal bacterium of the skin, it may have been transmitted via the diaper.

*T. bernardiae* is frequently reported in multiple bacterial infections. Therefore, when urinary tract infection occurs in the presence of several bacterial infections in old diaper-using patients with reduced ADL, such as in this case, it is necessary to note the presence of rare bacteria that can cause opportunistic infections.

Our study had some limitations. First, as this study is a case report, it is difficult to generalize the findings to other very old, diaper-using, bedridden patients with reduced ADL. In addition, the long-term health effects of *T. bernardiae* infection remain unknown. In the future, we hope to investigate the long-term health effects of *T. bernardiae* infection by conducting long-term observations of this patient population.

## Conclusion

We report a rare case of sepsis following acute pyelonephritis caused by *T. bernardiae.* Very old patients with reduced ADL who are bedridden and use diapers are prone to infections and chronic cystitis. When such patients present with urinary tract infections with multiple bacterial infections, we should consider infections caused by rare bacteria or opportunistic infections.

## Data Availability

All data generated or analyzed during this study are included in this published article.

## Abbreviations

ADL: Activities of daily living
GCS: Glasgow Coma Scale
WBC: White blood cell
CRP: C-reactive protein
SOFA: Sequential Organ Failure Assessment
qSOFA: Quick SOFA
MALDI-TOF MS: Matrix Assisted Laser Desorption/Ionization Time of Flight Mass Spectrometer
CLSI: Clinical and Laboratory Standards Institute
EUCAST: The European Committee on Antimicrobial Susceptibility Testing
S: Susceptible
R: Resistant
ABPC/SBT: Ampicillin/sulbactam
CEZ: Cefazolin
CEX: Cephalexin
